# Soundscapes of morality: Linking music preferences and moral values through lyrics and audio

**DOI:** 10.1371/journal.pone.0294402

**Published:** 2023-11-29

**Authors:** Vjosa Preniqi, Kyriaki Kalimeri, Charalampos Saitis

**Affiliations:** 1 Centre for Digital Music, Queen Mary University of London, London, United Kingdom; 2 ISI Foundation, Turin, Italy; Tokyo Institute of Technology: Tokyo Kogyo Daigaku, JAPAN

## Abstract

Music is a fundamental element in every culture, serving as a universal means of expressing our emotions, feelings, and beliefs. This work investigates the link between our moral values and musical choices through lyrics and audio analyses. We align the psychometric scores of 1,480 participants to acoustics and lyrics features obtained from the top 5 songs of their preferred music artists from Facebook Page Likes. We employ a variety of lyric text processing techniques, including lexicon-based approaches and BERT-based embeddings, to identify each song’s narrative, moral valence, attitude, and emotions. In addition, we extract both low- and high-level audio features to comprehend the encoded information in participants’ musical choices and improve the moral inferences. We propose a Machine Learning approach and assess the predictive power of lyrical and acoustic features separately and in a multimodal framework for predicting moral values. Results indicate that lyrics and audio features from the artists people like inform us about their morality. Though the most predictive features vary per moral value, the models that utilised a combination of lyrics and audio characteristics were the most successful in predicting moral values, outperforming the models that only used basic features such as user demographics, the popularity of the artists, and the number of likes per user. Audio features boosted the accuracy in the prediction of empathy and equality compared to textual features, while the opposite happened for hierarchy and tradition, where higher prediction scores were driven by lyrical features. This demonstrates the importance of both lyrics and audio features in capturing moral values. The insights gained from our study have a broad range of potential uses, including customising the music experience to meet individual needs, music rehabilitation, or even effective communication campaign crafting.

## Introduction

Music is one of the most fundamental forms of expression. As Victor Hugo once said, “Music expresses that which cannot be said and on which it is impossible to be silent”. It is a part of the holistic human experience influencing our emotions [1] and cognitive performance, such as thinking, reasoning, problem-solving, creativity, and mental flexibility [2]. Besides, research has shown that people often select music that aligns with their empathy levels [3] and personality needs [4–7] and enables them to express their values [8, 9]. Such knowledge has proven to be effective for music recommendation systems and their diversity [10].

For this work, we investigate the less-attended connection between moral values and music preferences. Personality alone may not fully explain our music preferences and listening habits. While personal values are inherent motivational goals, moral values mirror traits, opinions and judgements formed under the influence of society, culture, upbringing and religion, which bring people together in groups. On the other hand, music has been considered a mechanism for fostering social relationships and bonding [11, 12]. Thus people may enjoy specific types of music because they provide stimuli that match their morality-related needs.

Our research aims to investigate to what extent people’s musical choices reflect their moral worldviews. We hypothesise that both audio and lyrics features of the musical pieces people like are important proxies for inferring their moral values. Lyrics are essential in the music listening experience, as they communicate emotions [13] and convey messages related to the main societal issues like love, life, death, and political or religious concepts. Musical audio components, which comprise several abstraction levels from basic perceptual parameters (e.g., melody, tempo) to more complex aggregated features (e.g., timbre, valence), can predict music-evoked emotions [1]; viral songs [14]; and music similarity [15]. Yet audio features of songs are not used as often to predict music preferences. Some studies have used musical audio features to interpret genre-based preference dimensions (e.g., [4]), but direct links to personality traits and values have only recently started to be explored [7, 16].

Similarly, song lyrics have also received little attention in personality science (e.g., [16, 17]. In our previous work [18], we investigated the link between moral values and music preferences in terms of lyrics. Here we want to see how audio features perform in inferring moral values and whether we can improve our previous findings by combining lyrics and audio. More specifically, the two main questions we pose for this study are: To what extent are moral values reflected in the acoustical and lyrical content of one’s favourite songs? Which music characteristics (lyrics vs audio) are more impactful for moral inferences? Frequently, textual and audio features have been used together for classifying moods [19, 20] and musical genres [21, 22]. However, there is limited knowledge about how the combination of audio and lyrics may influence the association between psychological traits and music choices.

To address these questions, we used data from the LikeYouth project (https://likeyouth.org, where participants agreed to fill out psychometric surveys and share their Facebook Page Likes. We explored participants’ moral scores and their Likes on music artist Pages and integrated lyrics and audio components from the five most popular songs per artist. We analysed the lyrics’ sentiment, overarching narratives, moral valence and emotional loadings. For audio analysis, we extracted 12 main high-level audio features from Spotify and the auxiliary low-level audio features such as pitch and timbre. We then build regression models for predicting moral values using the extracted features. Results established that people’s musical preferences can, to some extent, predict their moral values, revealing a clear association between the listeners’ preferences for artists whose musical and lyrical content aligns with their moral worldviews.

Our work adds to the growing body of literature on the interplay between music and psychology. Information about a person’s moral worldviews is essential for psychologically aware music recommendation systems, which can contribute to listener wellbeing [23]. On a different note, communication professionals would benefit from understanding the relationship between individuals’ moral beliefs and music choices when selecting music to accompany campaigns for social good while communicating messages through music.

### Links of personality traits and personal values with music preferences

In music psychology, numerous studies investigated the relationship between music preferences and personality traits [3–5, 24] using Costa and McCrae’s Big5 model [25]. This model is widely accepted and implemented in research, and it depicts five intrinsic traits: Openness, Extraversion, Agreeableness, Neuroticism and Conscientiousness. Scientists in other fields have used this framework for predicting individuals’ work role performance [26], analysing the sense of humour [27] and inferring user movie preferences [28] and choices and behaviours on social media and digital platforms [29–31].

Personality and music associations were initially analysed via musical stimuli in a lab setting using musical styles (e.g., reflective and complex, upbeat, conventional, energetic [4]) that emerged from genre preferences. Later, researchers took advantage of the fast expansion of internet-based services and used naturally occurring views of musical preferences in online social media [6]. Even more sophisticated models for inferring personality dimensions emerged as a result of behavioural analysis from online music streaming platforms [7] and by implementing finer-grained content-level features including audio [32], and lyrics components [33]. Melchiorre and Schedl [32] explored correlations between listeners’ personality traits and music preferences utilising Spotify audio features (e.g., danceability, loudness, mode, key, valence). They found that individuals with high openness to experience prefer acoustic songs and instrumental music rather than loud and energetic music. While extroverts prefer music with high danceability and positive valence, which is in line with the stereotypical depiction of outgoing individuals. Regarding lyrics, some studies pointed at affinities between songwriters’ personality, mental health and their lyrical content [17, 34]. From the listeners’ point of view, studies showed that neurotic individuals prefer listening to musical pieces with more complex and less repetitive lyrics that express negative emotions [33, 35]. Whereas more conscientious individuals prefer songs with lyrics talking about achievements [33], but also about love [16].

In addition to personality, few music studies have considered the exploration of personal values (Schwartz Theory [36]), which represent broader goals that motivate and/or inhibit prosocial actions and behaviours [37]. These values define four universal dimensions, namely Openness to change (stimulation and self-direction), Conservation (conformity, tradition and security), Self-transcendence (benevolence and universalism) and Self-enhancement (achievement and power). In their study about music preference, values similarity and social attractions, Boer et al. [38] argued that social bonding through music is likely to occur via value similarity but not via similarity in personality traits. Gardikiotis and Baltzis [8] used personal values as proxies to predict people’s musical tastes. They showed that openness to change was highly associated with non-mainstream music, while self-transcendence was associated with sophisticated and complex music. Yet another study showed that individuals with self-enhancement values prefer contemporary music styles, and individuals with self-transcendence values prefer sophisticated music styles [39]. Furthermore, the authors pointed to a link between openness-to-change values and preference for music serving various personal and societal functions, while conservation values are negatively associated with these functions.

Even though studies have yielded positive results concerning music, personality affiliations and personal values, moral worldviews are an essential factor that deserves additional attention from the research body. Not only do moral foundations partially reflect more dispositional personality traits [40, 41], but they also have the potential to give profound insight into why people have the musical tastes they do and how these choices align with their ethical reasoning.

### Moral values: From social/political perspectives to music lenses

For this work, we operationalise moral values via the Moral Foundations Theory (MFT) [42], which describes the psychological ground of morality in terms of five innate foundations, formed by a two-faced scope between so-called virtues and vices. These foundations are *Care/Harm, Fairness/Cheating, Loyalty/Betrayal, Authority/Subversion*, and *Purity/Degradation*. These can further collapse into two superior foundations: *Individualising* (Care and Fairness), indicative of a more liberal perspective, and *Binding* (Purity, Authority and Loyalty), indicative of a more conservative outlook. As Hoover and colleagues [43] explained, the MFT framework offers the most diverse and well-rooted pluralistic model of moral values. Also, it is the only taxonomy of morality with developed term dictionaries. Several dictionary-based techniques were proposed to infer moral values within the text of tweets and social media posts, such as the Moral Foundations Dictionary [44, 45] and the MoralStrength lexicon [46].

Researchers have associated morality with judgements, opinions and attitudes towards complex situations such as charitable donations [43], climate change [47], vaccination [48, 49], interpersonal exposure [50], and politics [40, 41, 51, 52]. While more recent works in computational social science have demonstrated the inference of moral values with various human behavioural digital data, including gameplay [53, 54], smartphone usage and web browsing [55]. Some researchers have investigated the potential role of morality subcultures in mediating the connection between one’s nationality and preferences for movie and TV genres [56]. While others have explored moral foundations for classifying public policies [57].

Generally, moral foundations are considered a higher psychological construct than the more typically investigated personality traits [58]. Some studies have pointed to a connection between moral values and personality attributes. In a study about the association of personality with political ideologies and moral values, Lewis and colleagues [40] found that Individualising foundations were correlated with Agreeableness and Openness. Whereas, Binding foundations were associated with Low-Openness, Extroversion and Conscientiousness. Furthermore, the authors noticed that higher levels of Neuroticism predicted higher levels of both Binding and Individualising, while these two super-foundations influence political orientation in the opposite direction [40]. Similar findings were demonstrated by Hirsh and colleagues [41].

“Morality binds and blinds” writes Haidt [59]; it has a huge impact on the way a person makes decisions and evaluates circumstances. As stated in previous studies, moral rhetoric can unite, persuade and motivate people with similar values or beliefs. However, it is unlikely to be efficient if the same rhetoric is used on both sides (e.g. conservatives vs liberals) [60, 61]. Hence, researchers debated *Moral reframing* as a technique for framing different situations in a way that is consistent with one’s moral values. For instance, Feinberg and Willer [60] stated that same-sex marriage is likely to get more support from conservatives if framed in terms of patriotism rather than equality.

Similar rhetoric was also communicated via musical sounds and lyrics in social and political drives. Commonly, the songs used in successful campaigns contain uplifting sounds and lyrics that resonate with nations’ ideals, such as values of optimism and progress for a better future [62]. Music rhetoric has been used to advocate for what is perceived to be a necessary societal change [63], promote peace and unity [64], and raise awareness for marginalised groups [65]. These narratives are closely related to moral judgements and beliefs. Despite that, moral values have yet to be elaborated more by music scientists. There is limited research on how musical choices or specific songs may elicit strong emotional responses in individuals who prioritise, for instance, Care and Fairness compared to those who prioritise Authority, Purity, and Loyalty.

### Links between moral values and musical tastes

In terms of morality and music studies, an association between moral values, demographics, and musical genres was explored by Preniqi et al. [66] using ad-hoc survey data. This study found that people with higher levels of Binding foundations tend to like country and Christian music. Those with lower levels of Binding traits tend to favour music genres such as punk and hip-hop, where lyrics are known to challenge traditional values and the status quo [8]. Individualising foundations were more difficult to predict (cf. [55]). Furthermore, the authors explained that adding demographic information (e.g., age, gender, political views, education) improved MFT predictions only to a limited degree, showing the ability of music preferences alone to describe one’s moral values [66].

Ansani and colleagues [67] investigated whether listening to a musical piece can affect the harshness of moral judgement. The results of this study suggested that negative emotions enforced by certain types of music can worsen moral judgement. Although, that study did not rely on a psychometrically validated tool like the MFT [67].

The link between the lyrics component and listeners’ moral values has been explored only recently. For instance, Messick and Aranda [68] analysed the link between lyrical preferences, moral reasoning domains and personality traits. The authors found that moral values define a unique and significant part of the variance in the lyrics preferences of different metal music sub-genre that was not explained by personality traits alone. For instance, liking lyrics about celebrating metal culture and unity relates to higher levels of Loyalty foundation and Extroversion. Whereas favouring song lyrics linked to hardships, love, and emotional turmoil is related to care/harm and fairness, showing that those individuals might use these lyrics as coping mechanisms or be more empathetic to those who struggle.

In recent work, Preniqi et al. [18] investigated moral values inferences based on a set of textual features related to lyrics’ overarching narrative, moral valence, sentiment and songs’ word emotion associations. The study demonstrated that lyrics components extracted from the naturally emerging music preferences in social media, to some degree, allow for constructing reliable premises of moral values. People who value more Individualising values preferred artists whose songs’ lyrics talk about joy, anticipation and trust. Those concerned more about ingroup and Binding values liked artists whose songs contained more romantic topics and disliked lyrics that implied negative emotions such as sadness, fear, or disgust [18]. However, as in the previous studies [55, 66], the Individualising foundations remained challenging to predict. Here, we extend this work by adding a range of low- and high-level audio features separately and in a multimodal framework, together with the lyrics, to improve the present results further, building on Tagg’s socio-semantic view of musical meaning [69] and motivated by recent findings linking individual audio features of preferred music to personality traits and values [4, 7, 16].

## Materials and methods

### Data

We used existing data previously collected via the LikeYouth Facebook-hosted application (https://likeyouth.org). LikeYouth is an innovative surveying tool originally conceived and developed independently by researchers at the ISI Foundation in Turin, Italy, where the second author is based, to address different research questions from the analyses reported here [48, 70]. It was deployed in March 2016 mainly in Italy, where approximately 64,000 people entered the application. On the basis of voluntary and informed consent (see Ethical Considerations), respondents filled out several psychometric questionnaires, including MFT, and provided demographic information (e.g., age, gender) and access to Facebook Page Likes. Data were fully anonymised to ensure that individual users could not be identified. The data used in this study were downloaded in September 2019, focused on 3,880 users who filled out the MFT questionnaire correctly.

We filtered the data further by retaining only those participants who have liked 10 or more music artist Pages on Facebook. This process left us with 1,480 respondents for whom we initially retrieved the top 10 most popular songs for the music artists they liked. We presumed that if a user liked the Page of a specific artist on Facebook, then that artist’s most famous songs reflect the user’s music preferences. We considered only those songs for which we were able to obtain both lyrics through the Genius API (Application Programming Interface) and audio features via the Spotify API. This resulted in 47,580 songs. We then used the spaCy library [71] to identify and select only songs with English lyrics, because lyrical features were extracted using tools developed for English text. This left us with 36,902 songs. We conducted predictive tasks using different numbers of artists’ most popular songs (10, 5, and 3). We found that using the top 5 songs provided the best balance between predictions, computational resources, and within-musician variability in lyrical and audio content while maintaining a sufficient number of artists and songs. The final dataset thus comprised 5,464 artists and 27,320 songs.

In terms of demographics, because of how LikeYouth collected gender information, we were “forced” to use it as a binary variable (woman or man). We are aware that there are many more gender identities that prospective studies should consider. There were also missing data related to age as 47% of the users (in our dataset) did not provide it. We chose to predict the missing values from Facebook Page Likes. We devised a sparse matrix representation of Page Likes per user and applied SVD to reduce the dimensionality [6, 30]. We binned the age attribute (median = 25) into two groups of “younger” (< 25) and “older” (≥ 25), and used an XGBoost classification model to predict missing age values [55], which had an estimated AUROC of 0.79 with a standard deviation of 0.018. The breakdown of our sample according to gender and age is reported in S1 Table. To mitigate the potential for bias in our models, we ran the same experiments while only considering users who provided their ages. We found that the predictions were similar for the Binding but slightly lower for the Individualising foundations.

**Fig 1:**
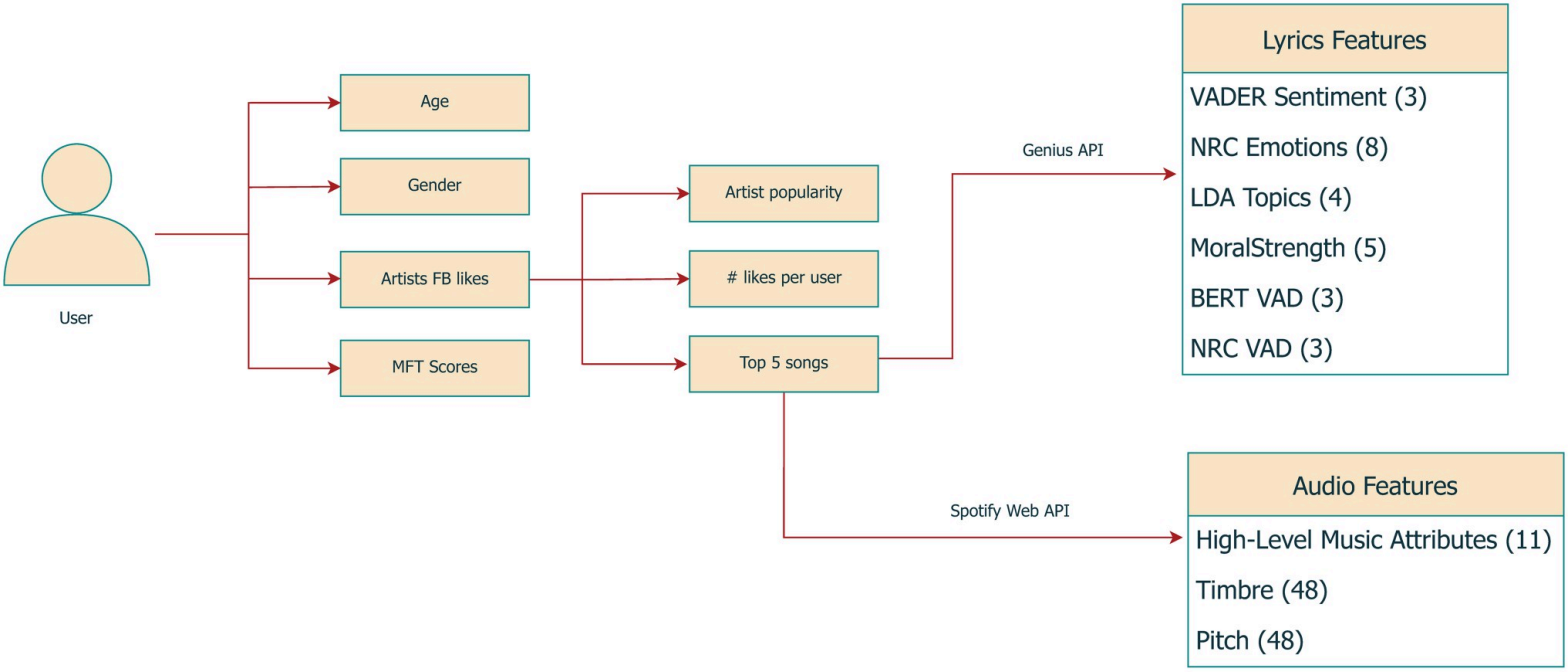
Overview of methodological framework.

Fig 1 depicts the framework we developed for this work. In total, we estimated 26 features for lyrics and 107 for audio (see subsequent sections). In addition to age and gender, baseline features included two additional (more shallow) digital traces as potential indicators of users’ music preferences. The first was the number of Page Likes per user (mean 35.11, standard deviation 33.95), and the second was a measure of artist popularity based on the number of followers on their Pages provided by LikeYouth. To our knowledge, LikeYouth is the only dataset that includes users’ moral values scores and a potential proxy of their music preferences.

### Ethical considerations

The LikeYouth app, including its Privacy Policy, was reviewed and approved by Facebook Inc. through the company’s official development process (https://developers.facebook.com/docs/app-review/). Data collection was fully anonymised and conducted in strict compliance with the terms and conditions set forth by the official Facebook API. Furthermore, the LikeYouth application had obtained authorisation from The Italian Data Protection Authority (https://www.garanteprivacy.it/). Consequently, data was obtained in a lawful and authorised manner, and in line with the principles expressed in the Declaration of Helsinki. All LikeYouth users, including those in our dataset, read and accepted the app’s Privacy Policy and explicitly gave access to their Facebook Likes.

### Lyrics content analysis

For each song, we extracted textual components related to its topic, moral valence, sentiment, and emotion. Then we used different text preprocessing approaches based on the corresponding feature modelling method. We applied traditional lexicon approaches and more advanced Natural Language Processing (NLP), such as BERT (Bidirectional Encoder Representations from Transformers) pretrained model. We performed minimal text cleaning and processing for sentiment detection with Vader lexicon and kept punctuation and capitalisation in the text. For the other lexicon methods, we employed the spaCy lemmatiser to extract Part of Speech lemmas from the lyrics. On average, each lyric contained 275 words and 109 lemmas.

#### Topic modelling

We applied a topic modelling approach based on Latent Dirichlet Allocation (LDA) [72] to discover common patterns in the lyrics narratives. LDA is one of the most popular methods for identifying latent thematic structures due to its simplicity, high accuracy in topic modelling and good computational efficiency [73]. The implementation of the LDA includes three main concepts: the text collection known as the corpus, individual text items known as documents, and the words within the documents referred to as terms [74]. In our case, the input of the LDA model is a term frequency matrix of the corpus created by our song lyrics data. To eliminate very general terms that may lead to irrelevant topics, we ignored terms with a frequency higher than 90%.

For an optimum number of topics *k*, we applied the topic coherency (*C*_*v*_ metric [75]) for models with *k* ∈ [2, 16] using a step size of 2. The experiments showed that *k* = 4 was the adequate number of topics for maximising the coherency. For *k* > 4 we obtained topics that were either generic or hard to distinguish due to the mixture of different words belonging to multiple topics, while for *k* < 4 we encountered topic ambiguities. The songs from our dataset talk primarily about (i) love and romance, (i) our everyday actions (narrating life/time related topics), (iii) vulgarity and obscene themes, and (iv) death, fear and violence. These four topics align well with previous work [76, 77]. Table 1 reports 10 representative songs per topic ranked by descending weight in the specific narrative. Because songs with large repetition (and sometimes with less content) tend to be at the top of the representation of the topic, we manually selected songs with high topic relevance and rich and varied content.

**Table 1:** LDA lyrics topics with example songs.

Topic	Artist	Song Title	%
Romance (0.34)	Mike Williams	Give it up	99
	Jody Watley	Real Love	98
	Jessie Andrews	I Never Knew	98
	Chris Lake	I Want You	98
	Cerrone	Give Me Love	97
	Charlie Puth	We Don’t Talk Anymore	97
	Cassie	Me & U	97
	The Chainsmokers	This Feeling	96
	Bebe Rexha	I Got You	96
	Cecile	Take My Heart	95
Time/Life (0.29)	Velvet Revolver	Fall to Pieces	97
	Unkle	Farewell	96
	Modstep	Rainbow	95
	Richie Sambora	One Light Burning	94
	Rebelion	City Lights	94
	Manse	Rising Sun	93
	Gabriel	Dresden Underwater	92
	Lake of Tears	So Fell Autumn Rain	91
	Sonic Boom	Just Imagine	91
	Noisecontrollers	The day	90
Profanity (0.20)	Tyga	Rack city	98
	The Sickest Squad	Sick lullaby	97
	Cardi B	Bartier Cardi	94
	Fat Joe	Yellow Tape	93
	Spice	So Mi Like It	93
	Skrillex	Dirty Vibe	92
	T-Pain	Dan Bilzerian	92
	Crypsis	The main mf	91
	Chamillionaire	Ridin’	88
	Wiz Khalifa	We Dem Boyz	88
Death/Fear/Violence (0.17)	Grand Magus	Forged in Iron—Crowned in Steel	96
	Brutality	Cries Of The Forsaken	95
	The Faceless	Xenochrist	95
	Krisiun	Scourge of the Enthroned	93
	Belphegor	Conjuring the Dead	92
	AsPphyxiate	Self Transform from Decayed Flesh	90
	Satanika	Steel Aggressor	88
	Destruction	Thrash till Death	89
	Dissection	Dark Mother Divine	87
	Kreator	Enemy of God	86

Brackets report overall topic prevalence. Songs ranked by topic proportion (%).

#### Moral valence

To analyse the moral valence of the lyrics, we used the MoralStrength lexicon [46], a tool that has a novel performance in predicting the moral valence within a text. This lexicon approach expands the Moral Foundation Dictionary (MFD) by offering three times more moral-annotated lemmas using the WordNet lexical database [78] and giving a set of normative ratings for empirical evaluation of morality that goes beyond the binary nature of MFD [46]. This lexicon includes a numeric assessment (moral valence score) of each lemma’s moral content. This score is based on a Likert scale from 1 to 9, with 5 considered neutral, reflecting the intensity and polarity of the lemma concerning the five moral foundations (MFT traits). Scores below 5 are associated with notions of harm, cheating, betrayal, subversion, and degradation, while scores above 5 are associated with care, fairness, loyalty, authority, and purity, respectively.

To determine the moral valence score for each song lyric, we calculated the average moral score of all the lemmas in the lyric. The moral foundation polarities do not directly translate as opposites (e.g., “not care” is not the same as “harm”); thus, the negation correction was not applied here. Because of the MoralStrength lexicon’s limited linguistic coverage, we could not infer moral valence for 20% of the collected lyrics. Instead, we annotated all the songs with undetected moral valence with the neutral point (five) of the Likert scale. Even though this approach pushed the observed mean towards the centre, it still captures the variability of the moral values across all the lyrics data. This technique allowed us to compare the moral valence expressed in the lyrics of the participants’ favourite artists with the moral values that the participants themselves reported on the questionnaires.

#### Sentiment and emotion analysis

We complemented the analysis of the lyrics by estimating their sentiment and emotional content. Although the two concepts have often been used interchangeably, sentiments are differentiated from emotions by the duration in which they are experienced [79]. Emotions in textual data represent temporary and preconscious phenomena described via assessment, physiological response, expressive display, feeling, or action tendency. In contrast, sentiments, which are lasting and conscious emotional tendencies, are usually classified as positive, negative, or neutral [79].

In this work, we utilised the commonly used VADER (Valence Aware Dictionary and sEntiment Reasoner) model [80] on the lyrics to acquire information about the sentiment of each song. The VADER model effectively analyses long and short texts and assigns positive, neutral, and negative sentiment scores. In addition to these basic categories, text often includes more nuanced expressions of emotion. [81]. Therefore, we also used the NRC Word-Emotion Association Lexicon [82] to evaluate the eight basic emotions described in the Plutchik wheel of emotions [83]. This lexicon has proven effective for analysing word emotion affinities in unlabeled textual data ([84]. We annotated each song’s lyrics with the eight emotions (anger, joy, sadness, fear, trust, anticipation, surprise and disgust) by averaging the word-emotion association scores.

Psychology studies often express emotion dynamics in a dimensional account of emotions described by two main dimensions, Valence and Arousal [81]. Sometimes a third dimension, Dominance, which portrays weak vs powerful, is included. We also used these three dimensions to further annotate lyrics with emotion dynamics by employing two practical approaches, VAD NRC Lexicon [85], and pre-trained BERT model [86]. The first approach presents human ratings of Valence, Arousal and Dominance for more than 20,000 English words [85]. For annotating the lyrics of each song based on these three dimensions, we averaged across words in the lyrics. This approach has limitations because the word annotation is independent of the whole sentence in the text and doesn’t consider the context.

We thus also considered BERT embeddings for the same task, which allow for multiple numeric representations for the same word based on the context. We fine-tuned a BERT classifier using the Musical Sentiment Dataset [87], which presents labelled songs for the affective dimensions (Valence, Arousal and Dominance). Since this dataset does not provide the lyrics of the songs, we extracted the lyrics of 40,000 English songs from this dataset using Genius API, querying them by the artist name and song title. We then predicted the Valence Arousal and Dominance (VAD) dimensions for our lyrics data using the pre-trained and fine-tuned BERT model. We compared the effectiveness of the two approaches by using them as predictors of participants’ self-reported moral scores (see the Experiments and Results section).

### Spotify audio features

We descibed audio characteristics of songs through three sets of track features provided by the Spotify API: (i) high-level music attributes, (ii) timbre features, and (iii) pitch features. For each user, we aggregated the audio features of the top 5 songs of their preferred artists.

#### High-level music attributes

This set included 11 Spotify audio features pertaining to a song’s general musical form, context, and mood: *energy* (perception of intensity and activity in a track); *danceability* (how suitable a track is for dancing); *valence* (probability that the track conveys positiveness); *tempo* (pace of the track in beats per minute or BPM); the *key* the track is in; *mode* (major or minor); average *loudness* of the track in decibels (dB); *instrumentalness* (probability that a track does not contain vocals); *speechiness* (presence of spoken words); *acousticness* (probability that a track is acoustic); and *liveness* (probability that the track was performed live). These features are computed for the entirety of a given song. Most are scalar between 0 and 1, except for key (categorical), mode (binary), tempo (BPM), and loudness (dB). More information can be found in the Spotify API documentation (https://developer.spotify.com/documentation/web-api/reference/get-several-audio-features).

#### Timbre

In its most basic, narrowest definition, musical timbre is broadly thought of as any property other than pitch, duration, and loudness that allows two tones to be qualitatively distinguished [88]. In the context of multitrack audio, such as songs, the notion of timbre can be expanded to refer to the “way it sounds,” that is the overall emerging timbre mixture of a disco mix, a jazz quartet, or a rock band [89]. The timbral content of a musical piece provides critical cues that enable listeners to categorise it according to genre and style [90] but also for conveying mood and emotion [91]. Spotify represents a song’s timbre as a vector of 12 unbounded values (timbre_1 to timbre_12) roughly centred around 0 for each song segment (the number of segments varies based on the song’s duration). We followed the approach of segment aggregation used in a previous work [14], which involves calculating various statistical moments for each timbre dimension. These included the average, standard deviation, mean of the first-order difference, and standard deviation of the first-order difference across all segments. This resulted in a total of 48 timbre features for each song.

To offer some intuitive interpretation of the analysed timbre dimensions, we looked at the top 100 songs that had the highest average values for each timbre dimension across segments. We saw that songs with hard rock, punk, grunge, and metal music had high values for timbre_1, _2, _9, and _12. Social and traditional music, like Harlem renaissance, vocal harmony, folk, and songs with nuances of jazz and blues, retained high values for timbre_3 and _6. Contemporary and dance music, including new romantic, house, funk, and techno exhibited high values for timbre_4. Mixed types of music, like modern classical musical pieces and songs with nuances of pop and rap exhibited high values for timbre_5 and _8. Popular genres such as pop, rock, and hip-hop were more predominant for timbre_10 and _11. While indie, alternative, new wave, and experimental music mainly showed high values for timbre_7.

#### Pitch

Another salient perceptual dimension of music is pitch. When listening to a musical piece, we perceive the pitches (or pitch classes) of successive notes (C, C♯, D, etc.) and how they relate to each other (e.g., intervals, contours, chords). Pitch information therefore pertains to a song’s melodic and harmonic structure [92], and as such also provides important cues for emotions expressed in or elicited by music [93]. Similarly to timbre, Spotify represents the pitch content of an audio track’s segment by a 12-dimensional vector (pitch_1 to pitch_12) corresponding to the 12 pitch classes of (Western) music (pitch_1 is C, pitch_2 is C♯, … pitch_12 is B). Each pitch dimension has values between 0 and 1 that describe its relative dominance in the segment. For example, a C-major chord would likely be represented by large values of C, E, and G. We followed the same approach of segment aggregation as with the timbre vectors, which resulted in a total of 48 pitch features for each song.

### Prediction models and SHAP values

We conducted a range of experimental designs for predicting the moral values of the participants based on the lyrics and audio features extracted from their music preferences (the full list of experiments is given in S2 Table). We operated four supervised regression models, Support Vector Regressor, Random Forest, XGBoost, and ElasticNet, to predict moral values using a multivariate approach over a 10-fold cross-validation setting. Like in previous studies [7, 32], we aggregated and scaled all the independent features for each participant before feeding those to the regression models. We reported only the outcomes from the Random Forest as it was the best-performing model. As a metric for performance evaluation, we used Pearson’s correlation coefficients between the predicted and actual moral values scores. This metric was commonly used in previous research on predicting psychological traits based on music preferences [6, 7, 18].

We built prediction models using each set of lyrical and audio features. Then we combined and analysed these features’ impact on the proposed models. For that, we assessed SHAP values to understand our models’ general behaviour and the importance of each feature. SHAP (SHapley Additive exPlanations) is a game theory approach devised to illustrate the features’ contribution to the final outcome of any machine learning model [94]. SHAP values present both global and local interpretability, meaning that we can assess how much each predictor and observation contribute to the regressor’s performance.

## Results

### Correlation

**Fig 2:**
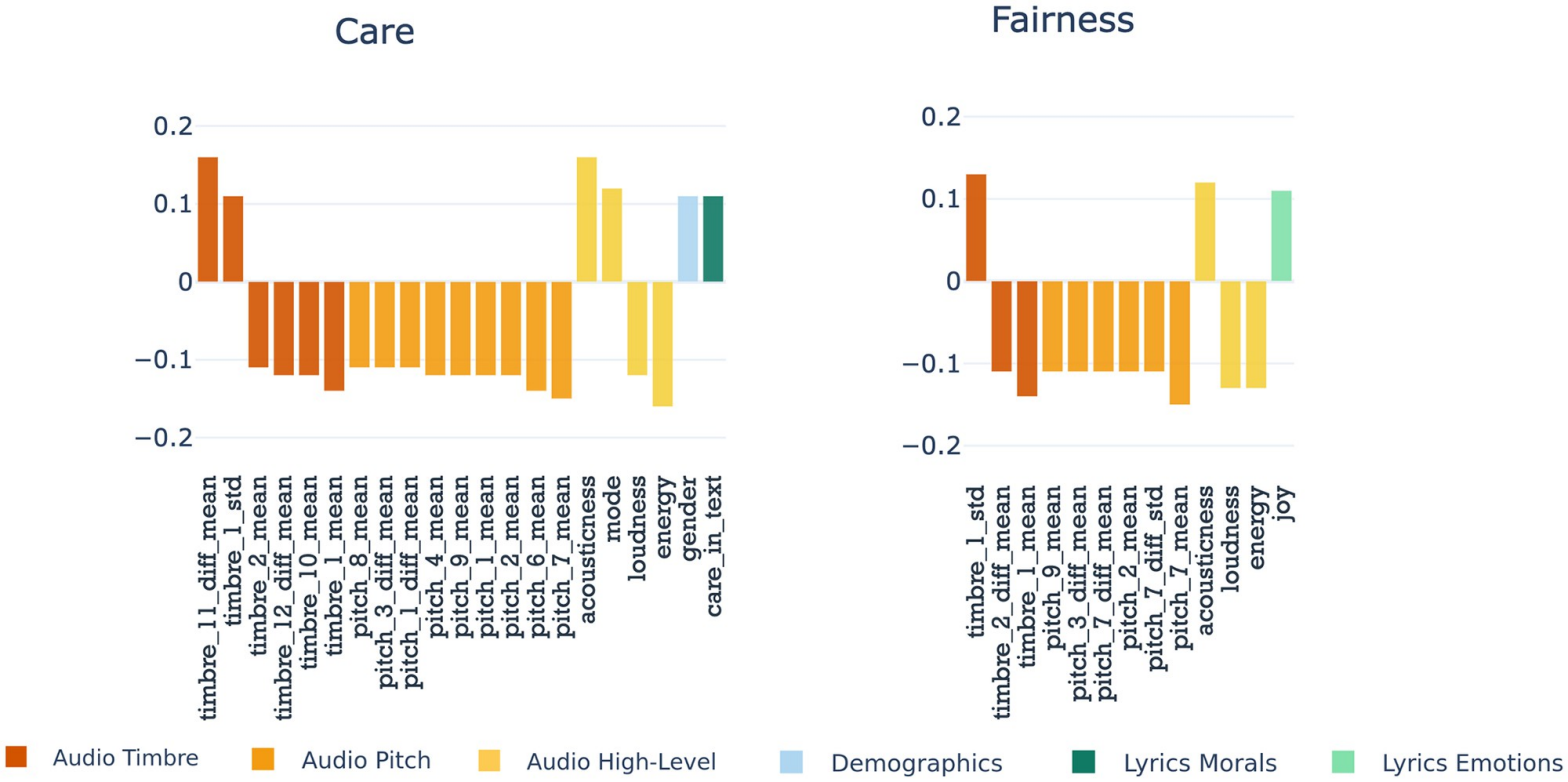
Significant Spearman correlations (*ps* < 5.3 x 10^−5^) for Individualising moral foundations.

**Fig 3:**
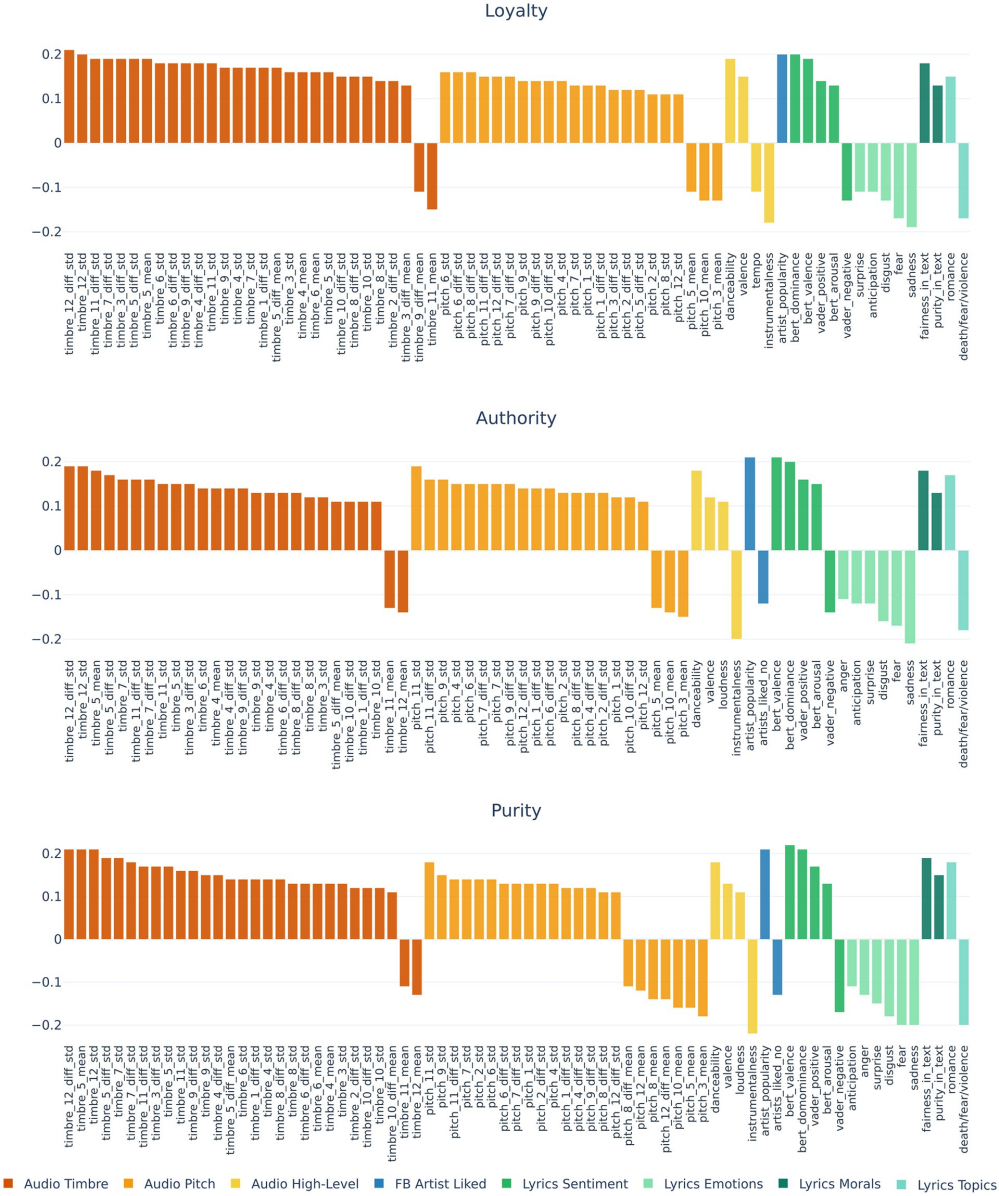
Significant Spearman correlations (*ps* < 5.3 x 10^−5^) for Binding moral foundations.

We explored the association between users’ moral values from the self-reported questionnaires, demographic attributes, and music preferences regarding lyrics and audio components. Figs 2 and 3 present the significant correlations between MFT values and lyrics and audio features for the five moral foundations, with a Bonferroni-corrected significance threshold (*ps* < 5.3 x 10^−5^) for each correlation coefficient. Similar plots for the Individualising and Binding super-foundations are given in S1 Fig. Complete correlations are reported in S2–S4 Figs. From the perspective of the lyrics’ linguistic cues, we saw that people who value more foundations related to Care and Fairness (Individualising values) prefer artists whose songs’ textual content is about care and joy.

Those concerned more about Loyalty, Authority and Purity (Binding or ingroup) foundations tend to choose artists whose songs’ lyrics talk about fairness, sanctity, and love. Also, individuals with strong ingroup values tend to prefer artists whose lyrics have positive sentiments and talk about dominance. This is intelligible as individuals who value Binding and their social groups tend to engage in group activities such as sports, religious events, and political gatherings, which often make use of music to promote messages of power, unity, and victory (e.g. sports chants, church choirs, etc.). On the other hand, participants with high Binding scores tend to dislike songs with negative valence, violent narratives and songs that resonate with sadness, fear, and disgust.

From an audio perspective, we saw that participants with Binding values preferred more artists whose songs are danceable, loud and with more positive sounds. In contrast, participants with Individualising values chose more artists whose songs are smooth, acoustic and have less dynamic sounds. Based on the correlation and statistical significance, timbre and pitch classes displayed important information about the link between individuals’ music choices and moral values. Binding foundations showed higher correlations across timbre and pitch dimensions, whereas Individualising foundations showed higher correlations to pitch classes. Individualising values showed a somewhat negative correlation to the dominant pitches (with values closer to 1), implying that people who value Care and Fairness prefer smoother rather than louder and dynamic music, which is in line with the findings from the higher-level musical features. Binding values had mostly positive correlations with timbre dimensions; however, it only showed a negative correlation with the averaged timbre 11 and 12 and the averaged first-order difference of time 9. We found that these particular timbre dimensions were dominant in hard rock, metal indie, pop, and electronic music (see Timbre subsection in the Materials and Methods section). This implies that individuals who value Loyalty, Authority, and Purity might prefer artists with more conventional and rhythmic songs and dislike those with rebellious, loud, distorted songs.

### Classification

For each moral foundation, we trained several models based on individual types of features as well as unimodal and multimodal combinations of features (see S3 Table). We also trained models with only the most meaningful features based on SHAP values. We used analysis of variance (ANOVA) to compare the significance of these “best features” models to corresponding “all features” models (see S4 Table). According to the ANOVA results, for Individualising values, the models constructed with all feature combinations were not significantly different from those with only the best features. However, the models constructed with all predictors were significantly better for Binding values than those with only the best features. As a baseline, finally, we set a model that includes features directly related to participants, such as demographics, the number of artist page likes per user, and artist popularity. Table 2 reports the baseline and best-performing model for each moral foundation.

**Table 2:** Best models for predicting moral foundations.

Moral Foundation	Baseline model	Best model	Features
Care	0.08 [-0.09, 0.23]	0.17 [0.01, 0.32]	all lyrics + all audio
Fairness	0.09 [-0.07, 0.25]	0.13 [-0.03, 0.29]	best audio + best lyrics
Loyalty	0.19 [0.03, 0.34]	0.23 [0.07, 0.38]	best lyrics + best audio
Authority	0.21 [0.05, 0.35]	0.28 [0.13, 0.43]	all lyrics + all audio
Purity	0.20 [0.04, 0.35]	0.29 [0.13, 0.43]	all lyrics + all audio
Individualising	0.10 [-0.07, 0.25]	0.18 [0.02, 0.33]	best lyrics + best audio
Binding	0.21 [0.06, 0.37]	0.33 [0.16, 0.46]	all lyrics + all audio

Pearson correlations (averaged across 10-fold cross-validation) between predicted values from regression and actual values. Brackets report 95% confidence intervals. “Best” features were obtained via SHAP feature importance models, see text for details.

According to the moral prediction results in recent studies, foundations related to Care and Fairness were challenging to predict [18, 66]. Our results again showed that moral values related to Care and Fairness are more complex to predict than values related to Authority, Purity, and Loyalty. However, when audio features were included, the prediction models for Individualising values showed significant improvement compared to the results of models trained with only lyrics features [18]. In contrast, the models for predicting Binding values only showed slight improvement when acoustic attributes were used in addition to lyrics features.

**Fig 4:**
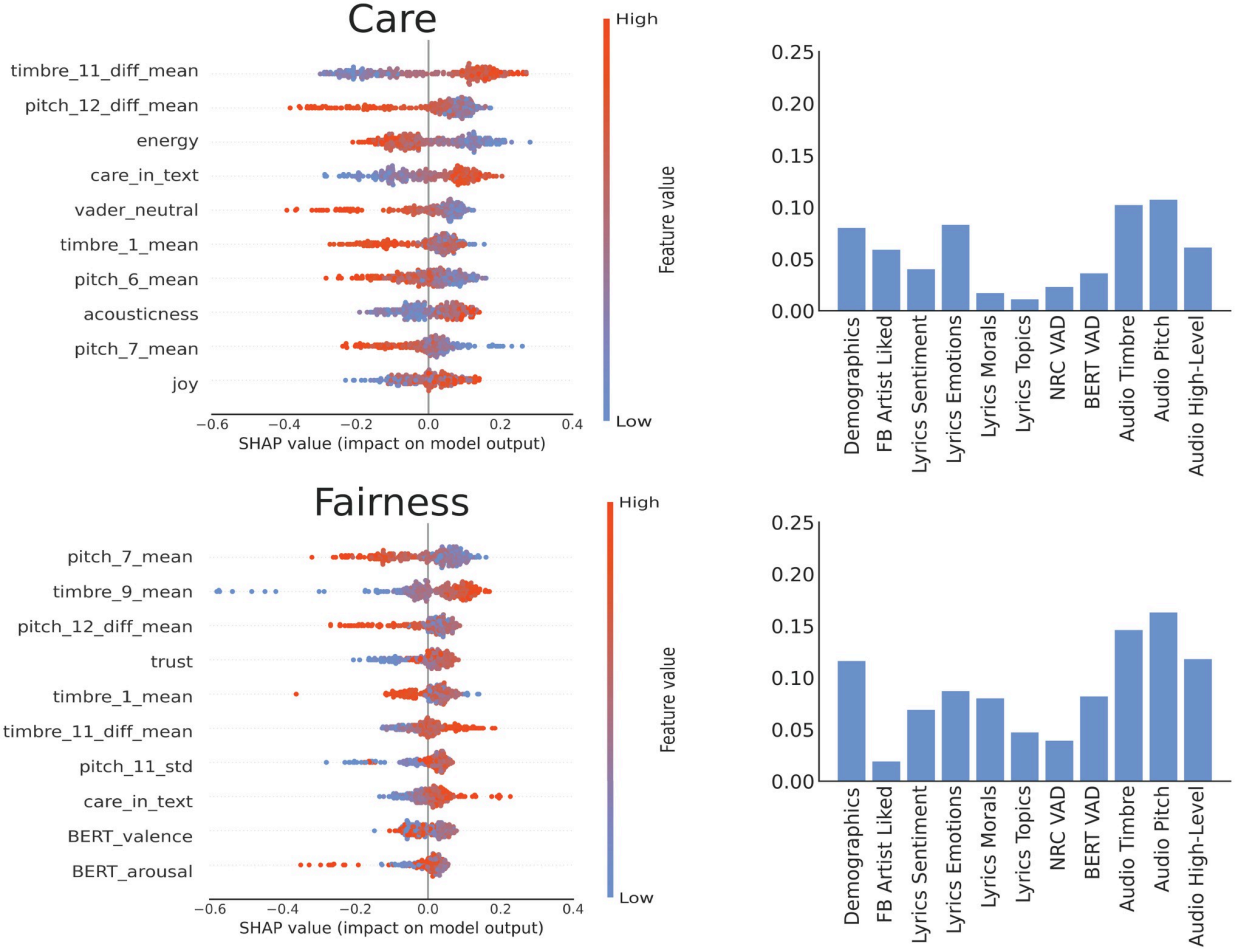
Feature importance for Individualising moral foundations. Left: Top 10 SHAP values from the model with best lyrics and best audio features. Right: Pearson correlations between predicted values from regression and actual values for different groups of features.

**Fig 5:**
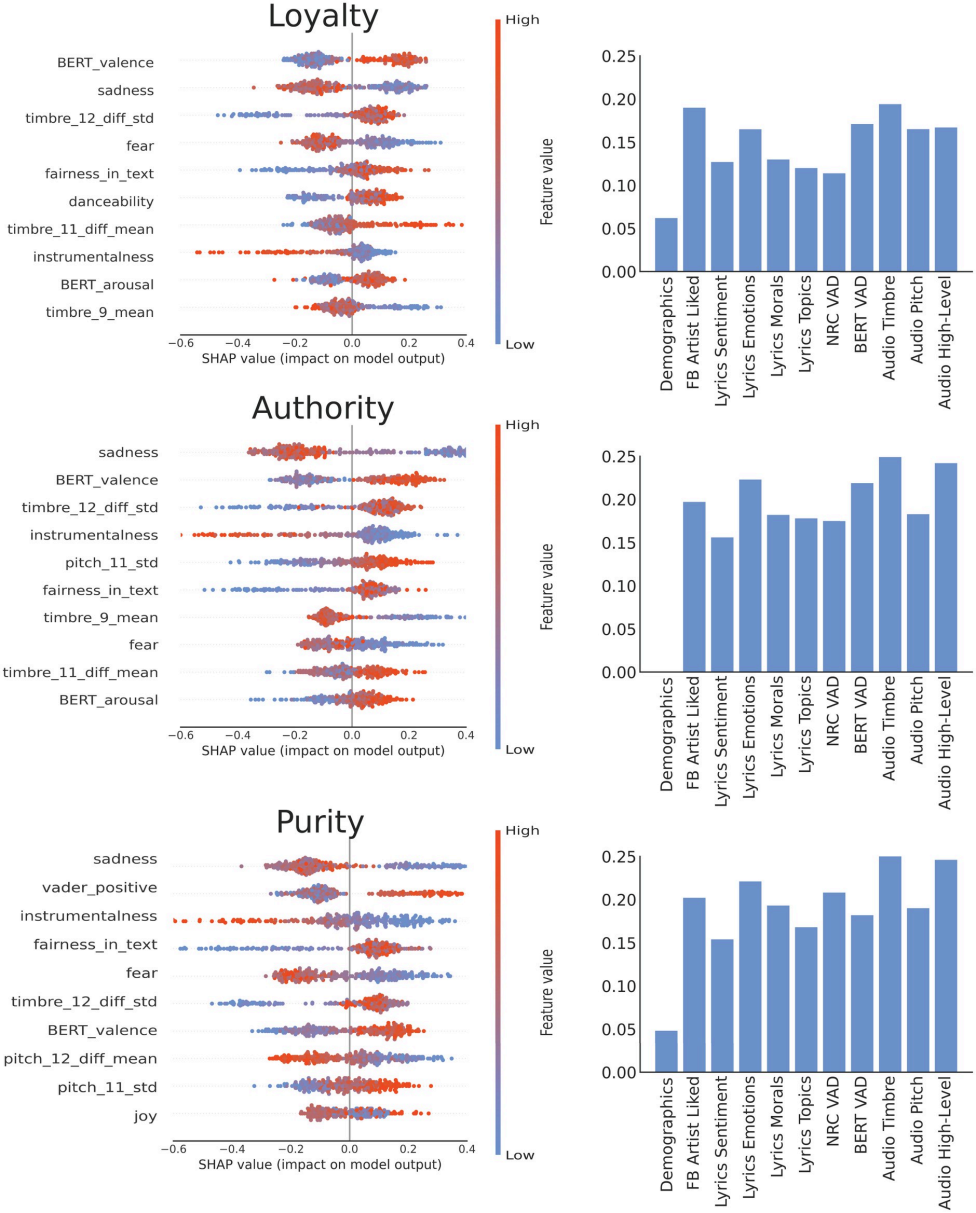
Feature importance for Binding moral foundations. Left: Top 10 SHAP values from the model with best lyrics and best audio features. Right: Pearson correlations between predicted values from regression and actual values for different groups of features.

The left panels in Figs 4 and 5 depict the 10 most important individual features for predicting each of the five moral foundations. These are based on SHAP values from the model with best lyrics and best audio features. Corresponding plots for the Individualising and Binding super-foundations are provided in S5 Fig. In line with observed correlations, feature importance representations for regression models show that for Care and Fairness audio features linked to pitch and timbre tend to outperform lyrical features associated with overall positive emotions such as joy and trust. In contrast, stronger and more opposite polarities of lyrics sentiment and emotions account for better predictions of Loyalty, Authority and Purity (e.g., fear, sadness, positive and negative valence). The importance of lyrics for these moral traits may be further reflected in their negative correlation with the instrumentalness of songs, that is, those who value more binding traits tend to prefer music with vocals rather than without.

For predicting Care and Fairness, the models constructed using pitch and timbre moments were the most effective among those built with isolated groups of features, followed by models built with demographic and emotion attributes (see right panel of Fig 4). When predicting Loyalty, Authority and Purity, the models built with high-level acoustic features, timbre moments, word emotions, and lyrics-BERT emotion valence were the most effective among those built with isolated groups of features, followed by models built with moral valence and topics (see right panel of Fig 5). The BERT models for lyrics sentiment slightly outperformed models with the same attributes acquired by the lexicon model, showing the importance of context in BERT embeddings for estimating the emotional valence of textual content like lyrics.

Overall, models built with compound features almost consistently outperformed the models constructed with one group of predictors (e.g. only valence features or only topics). These models also outperformed the baseline model built with demographic characteristics (significant for Individualising values), the number of likes per user and artist popularity (significant predictor for Binding values). Moreover, combined lyrics features were more effective predictors of moral foundations related to ingroup Binding. In comparison, integrated audio features were better predictors of moral foundations related to Individualising.

## Discussion

This study explored the relationship between a person’s morals and the type of music they prefer, considering both the songs’ lyrics and acoustic features. In addition, we investigated the influence of demographics and shallow digital traces, such as artist page likes per user and artist popularity, in inferring an individual’s moral foundations. From the results of MFT questionnaires, we saw that women scored higher than men in Care, Fairness and Purity foundations. We also found that individuals with more central Binding and Ingroup values preferred more popular and mainstream artists suggesting that these individuals are more likely to be influenced by social norms and group opinions. This is consistent with the idea that music preferences play a role in social bonding, as shared musical tastes can indicate similar values and dispositions [95].

We examined various methods for analysing the lyrics of songs, including more traditional techniques such as lexicons and more complex natural language processing methods like the BERT model. We complemented the moral and lyrics analysis presented previously [18] by analysing high-level and low-level acoustic characteristics. Previously, we found that by analysing the lyrics of one’s preferred artists, reliable inferences about that individual’s moral values can be made [18]. Values associated with ethics of tradition and hierarchy were more accurately predicted by lyrics features than values related to virtues of equality and compassion.

Building on Tagg’s socio-semantic view of musical meaning [69], we hypothesised that audio features play an important role in comprehending how people align their music preferences with moral worldviews. Despite difficulties in predicting Care and Fairness foundations reported previously [18, 66], we improved the predicting results for these foundations mainly when adding acoustic features. This was in line with correlation results which suggested that individuals with higher scores for Care and Fairness preferred artists whose songs are more acoustic and include fewer speech elements. While for Authority, Purity, and Loyalty, the prediction results with audio features improved only marginally. They did not outperform lyrics predictors, implying that individuals who value these foundations more may prioritise lyrics that align with their beliefs over the song’s musicality when selecting music to listen to.

Further, we found that models built with either timbre or pitch features had good accuracy for predicting each moral foundation. One aspect that could have led to the high accuracy of these models is the inclusion of a large number of attributes through statistical moments (mean, standard deviation, mean of first-order difference, and standard deviation of first-order difference) calculated for every dimension of pitch and timbre. This allowed us to gain a more in-depth comprehension of the information encoded in the musical compositions. Even though models built with lyrics features expressing emotions, morals, and sentiments employed significantly fewer attributes, they too demonstrated a decent accuracy in predicting overall moral proportion.

Our experimentation showed that both the lyrics and audio features of songs play significant roles in describing individuals’ musical preferences. Moreover, the most accurate predictions of moral values were obtained when considering both lyrics and audio elements within a multimodal framework. From a general interpretation, our findings indicated that participants with higher Individualising scores tended to prefer music with smooth, low-arousal melodies and lyrics that express themes of trust, anticipation, and care. On the other hand, those with higher Binding scores tended to prefer more upbeat, rhythmic songs with lyrics that focus on themes of love, purity, and victory. Given the correlation between moral values and political leanings [40], these broad implications might have some similarities with Rentfrow and Gosling’s [4] results, which suggested that more liberal individuals preferred reflective and complex music (e.g. jazz, blues, and classical), while more traditional and conservative individuals preferred more traditional and upbeat music (e.g. pop and religious music).

Even though we showed improvements in predicting Individualising foundations (.10 ≤ *r* ≤ .18) by 7% compared to the previous study [18], models predicting Binding values were more successful (.21 ≤ *r* ≤ .33), suggesting that binders more instinctively express their moral values through their musical choices. The results are encouraging but still fall short of reaching the desired level. The nature of the moral values being analysed is highly complex, and such attributes are often conveyed through subtle means daily and occasionally in a more intense way under certain circumstances [55].

Therefore, plenty of research literature uses personality traits as a more trivial proxy for analysing the implications of musical tastes. For instance, also using music-related Facebook likes, Nave et al. [6] obtained Pearson correlations in the range of .16–.30 on their regression models for the prediction of Big-Five traits. Anderson et al. [7] achieved Big-Five inferences in the range of .26–.37 by using a more complex framework using musical moods, genres and other derived metrics derived from music listening behaviours on the Spotify platform. Our results are comparable to these studies, corroborating our motivation that moral reasoning styles are also vital in understanding musical preferences [68]. Our findings help to further increase awareness about how our music listening habits may reveal information about our values and worldviews.

Prospective studies should further examine the relationship between moral worldviews and music, particularly in cross-cultural contexts, as these psychological factors are crucial in the decision-making process in various societal issues process [55, 96]. In his book *The Righteous Mind* [59], Jonathan Haidt explained that Republicans have a better understanding of moral psychology and how it is used in political campaigns, as evidenced by their ability to use moral rhetoric in slogans and songs effectively by triggering the full range of intuitions described by the five MFT traits. Like Democrats, they can talk about innocent people and fairness, but they put more emphasis on resonating with the virtues of loyalty, authority and purity; in contrast, Democrats only focus on the ethics of empathy and equality. Caiani and Padoan [97] provide a similar account of the multiple mechanisms linking the production of pop music (as a form of popular culture) to the rise and reproduction of populism Music chosen for social and political campaigns may therefore be attuned based on the targeted audience. Our findings can serve as a guide for future research in understanding how music can be used to stimulate and influence people in social and political contexts.

Another interesting aspect would be analysing moral values as a proxy, beyond demographics, to enhance the music listening experience and improve recommendations further. Traditionally, music recommendation systems mainly relied on statistical models, learning from historical user preference data. More sophisticated recommendation algorithms measured consumption diversity, and the interplay between users’ short-term and long-term engagement with a particular streaming platform [98]. Nonetheless, music streaming services have yet to consider the psychological and emotional state of the listener. Therefore, we believe that the insights gained from our work can be incorporated into music recommendation systems that are attuned to human psychological aspects. The application of these insights can be extended beyond music to other forms of media.

A critical aspect of this discussion is the ethical considerations when AI/ML systems are involved in predicting individuals’ demographics, psychological traits, music-listening habits, and preferences. Although this can be beneficial for campaigning in social and political contexts and enhancing recommendation systems, it can also be dangerous as it could be exploited for harmful purposes such as manipulation and control. To mitigate these issues, researchers and AI engineers need to share ethical usage guidelines for the deployed systems/platforms that can help users understand how their data will be used and the beneficial purposes of the data utilisation in these platforms. This way, users will have the opportunity to know how these systems work and decide whether they want to use them. Studies like ours can be used to raise awareness and increase public education about the potential AI/ML models to determine users’ preferences and psychological aspects based on their behavioural patterns.

Furthermore, the algorithms used to analyse the data from digital music streaming platforms may be biased against certain groups of people who have informed their demographics or particular worldviews, which could lead to unfairness and discrimination [99, 100]. While user feedback can sometimes homogenise recommendations over time [101]. Therefore, it is essential to ensure that any analysis related to personal characteristics on digital streaming platforms is conducted transparently, with proper mechanisms in place that mitigate any potential biases related to user interaction, social attitudes, behavioural patterns, sampling, population and data representation [99].

Our work has a few limitations. We crafted musical preference profiles from Facebook Page Likes which represent static data and may only reflect a snapshot of users’ music interests at a particular point in time. Digital streaming platforms could provide more detailed information about users’ music listening habits and patterns [7, 16, 102]. Furthermore, the LikeYouth project data include primarily Facebook users from Italy, so our conclusions may only partially apply to other cultures across the world. A related issue concerns the analysis of English-lyrics songs only. More specifically, because the LikeYouth app was launched primarily through the Italian language version of Facebook, the majority of the users who responded are expected to be *geolocated* in Italy, but this may not necessarily reflect *culturally* Italian users. Conversely, it is plausible that a user of Italian nationality geolocated (at the time of data collection) outside Italy may still use the Italian version of Facebook. The fact that our initial dataset of 47,580 songs comprised about 78% English lyrics indicates that our sample nonetheless had a very strong preference for English-speaking songs overall. This preference implies that English music holds a meaningful place in their regular listening habits, rather than being a random or occasional choice.

It should also be noted that the audio features available from the Spotify API are based on non-public algorithms. As such, it is not possible to quantify exactly which acoustical and musical parameters contribute to a particular audio feature. Future work will focus on custom feature extraction techniques based on existing open-source toolboxes for music content description [103]. Deep learning approaches for representing lyrical and audio content might also be of interest, for example, using state-of-the-art pre-trained audio embeddings for timbre [104] and BERT embeddings for topic modelling and moral valence [86] On a final note, although our results indicated that music preferences carry information about individuals’ moral worldviews that goes beyond basic demographics, there may be other factors not included in our study, such as socio-economic status, religion, culture, and hobbies, that could further support our findings.

## Conclusion

The association between an individual’s psychological traits and music listening preferences is becoming increasingly popular, particularly for customizing music recommendations, enhancing communication strategies, and advancing music therapy approaches for health and wellbeing. Different from widely discussed personality models, the more understudied moral values shape an individual’s psychological perspective, significantly impacting their decision-making and social situation evaluation. Here we reported results from an ensemble of experiments that investigated the degree of moral values’ predictability from song lyrics and audio features. We aligned psychometric moral values scores of 1,480 users to lyrics and audio features from the top 5 songs of their preferred music artists as emerged from Facebook Page Likes. We employed various techniques to identify the moral valence, attitude, and emotions conveyed in the lyrics. We also examined low- and high-level audio features provided via Spotify’s API. We found audio features to be better than lyrical features in inferring values of empathy and equality but only marginally so for values of tradition and hierarchy. Multimodal models were the most effective in predicting moral values. Inferring such worldviews from music preferences offers a way of sensing psychological and behavioural facets of societies at a large scale.

## Supporting information

S1 Table Demographic breakdown of our data according to gender and age. (PDF)

S2 Table Summary of experimental designs built for regression models with type and number (#) of independent features for each model. (PDF)

S3 Table Complete classification results. Pearson correlations (averaged across 10-fold cross-validation) between predicted values from regression and actual values. Brackets report 95% confidence intervals.
(PDF)

S4 Table Model significance (ANOVA) between “all” and “best” feature models. (PDF)

S1 Fig Significant correlations (*ps* < 5.3 x 10^−5^) for the Individualising and Binding moral super-foundations. (TIFF)

S2 Fig Feature importance for the Individualising and Binding moral super-foundations. Left: Top 10 SHAP values from the model with best lyrics and best audio features. Right: Pearson correlations between predicted values from regression and actual values for different groups of features.
(TIFF)

S3 Fig Complete Spearman correlations between MFT traits and lyrics features (top and middle panels) and high-level music attributes (bottom panel). Significance threshold (*) corrected to 5.3 × 10^−5^.
(TIFF)

S4 Fig Complete Spearman correlations between MFT traits and timbre features. Significance threshold (*) corrected to 5.3 × 10^−5^.
(TIFF)

S5 Fig Complete Spearman correlations between MFT traits and pitch features. Significance threshold (*) corrected to 5.3 × 10^−5^.
(TIFF)
