# Supplementary material for: Soundscapes of morality: Linking music preferences and moral values through lyrics and audio
Source: PLoS One. 2023 Nov 29;18(11):e0294402. doi: 10.1371/journal.pone.0294402 (PMC10686442; doi:10.1371/journal.pone.0294402)
Supplement: S1 Table — (PDF) [file pone.0294402.s001.pdf]

S1 Table

		All data $N = 3,880$	≥10 Page Likes $N = 1,480$
Gender	M	53%	53%
	F	47%	47%
Age	<25	21%	28%
	≥25	79%	72%
