# Supplementary material for: Soundscapes of morality: Linking music preferences and moral values through lyrics and audio
Source: PLoS One. 2023 Nov 29;18(11):e0294402. doi: 10.1371/journal.pone.0294402 (PMC10686442; doi:10.1371/journal.pone.0294402)
Supplement: S2 Table — (PDF) [file pone.0294402.s002.pdf]

S2 Table

Model	Features	#
	Segregated Sets of Features	
EX0	Baseline: Age & Gender & Artist Popularity & No. of Artist Likes per user	4
EX1	VADER Sentiment	3
EX2	NRC Emotions	8
EX3	MoralStrength Lexicon	5
EX4	LDA Topics	4
EX5	NRC VAD	3
EX6	BERT VAD	3
EX7	Timbre	48
EX8	Pitch	48
EX9	High Level Music Attributes	11
	Combination of Audio Features	
EX10	All audio Features	107
EX11	All audio Features & Baseline	111
EX12	Best audio Features	13
EX13	Best audio Features & Baseline	17
	Combination of Lyrics Features	
EX14	All Lyrics Features	23
EX15	All Lyrics Features & Baseline	27
EX16	Best of Lyrics Features	12
EX17	Best of Lyrics Features & Baseline	16
	Combination of Lyrics & Audio Features	
EX18	All Lyrics and Audio Features	130
EX19	All Lyrics and Audio Features & Baseline	134
EX20	Best Audio and Lyrics Features	25
EX21	Best Audio and Lyrics Features & Baseline	30
