# Supplementary material for: Soundscapes of morality: Linking music preferences and moral values through lyrics and audio
Source: PLoS One. 2023 Nov 29;18(11):e0294402. doi: 10.1371/journal.pone.0294402 (PMC10686442; doi:10.1371/journal.pone.0294402)
Supplement: S3 Table — Pearson correlations (averaged across 10-fold cross-validation) between predicted values from regression and actual values. Brackets report 95% confidence intervals. (PDF) [file pone.0294402.s003.pdf]

S3 Table

Models Built with Segregated Sets of Lyrics Features			
MFT	EX1	EX2	EX3
Care	0.06 [-0.10, 0.22]	0.08 [-0.08, 0.24]	0.08 [-0.08, 0.23]
Fairness	0.05 [-0.11, 0.21]	0.10 [-0.06, 0.26]	0.01 [-0.15, 0.17]
Loyalty	0.11 [-0.06, 0.26]	0.17 [0.01, 0.33]	0.12 [-0.04, 0.28]
Authority	0.14 [-0.02, 0.29]	0.22 [0.07, 0.37]	0.19 [0.03, 0.34]
Purity	0.15 [-0.01, 0.31]	0.21 [0.05, 0.36]	0.19 [0.03, 0.34]
Individ.	0.07 [-0.09, 0.22]	0.09 [-0.07, 0.24]	0.05 [-0.11, 0.21]
Binding	0.17 [0.01, 0.32]	0.24 [0.09, 0.39]	0.21 [0.06, 0.36]
MFT	EX4	EX5	EX6
Care	0.05 [-0.12, 0.20]	0.04 [-0.12, 0.20]	0.08 [-0.09, 0.23]
Fairness	0.01 [-0.16, 0.16]	0.02 [-0.14, 0.18]	0.05 [-0.11, 0.21]
Loyalty	0.11 [-0.05, 0.26]	0.11 [-0.05, 0.27]	0.18 [0.02, 0.33]
Authority	0.18 [0.02, 0.33]	0.18 [0.02, 0.33]	0.22 [0.06, 0.37]
Purity	0.17 [0.01, 0.32]	0.21 [0.05, 0.35]	0.18 [0.02, 0.34]
Individ.	0.05 [-0.11, 0.21]	0.04 [-0.12, 0.20]	0.07 [-0.09, 0.23]
Binding	0.18 [0.02, 0.33]	0.21 [0.05, 0.36]	0.23 [0.07, 0.38]

(continued on next page)

S3 Table (continued)

Models built with Segregated Sets of Audio Features				
MFT	EX7	EX8	EX9	
Care	0.14 [-0.02, 0.29]	0.15 [-0.02, 0.30]	0.11 [-0.05, 0.26]	
Fairness	0.12 [-0.04, 0.27]	0.11 [-0.05, 0.26]	0.06 [-0.10, 0.22]	
Loyalty	0.19 [0.03, 0.34]	0.16 [-0.00, 0.31]	0.17 [0.01, 0.32]	
Authority	0.25 [0.10, 0.40]	0.19 [0.03, 0.34]	0.25 [0.09, 0.39]	
Purity	0.25 [0.09, 0.39]	0.19 [0.03, 0.34]	0.24 [0.09, 0.39]	
Individ.	0.14 [-0.02, 0.30]	0.17 [0.01, 0.32]	0.11 [-0.05, 0.26]	
Binding	0.28 [0.13, 0.42]	0.21 [0.05, 0.36]	0.27 [0.11, 0.41]	
**Models built with Combination of Audio Features**				
MFT	EX10	EX11	EX12	EX13
Care	0.16 [0.00, 0.31]	0.17 [0.01, 0.32]	0.15 [-0.01, 0.30]	0.15 [-0.01, 0.31]
Fairness	0.12 [-0.04, 0.28]	0.12 [-0.05, 0.27]	0.13 [-0.03, 0.29]	0.13 [-0.03, 0.29]
Loyalty	0.20 [0.04, 0.35]	0.21 [0.05, 0.36]	0.20 [0.04, 0.35]	0.21 [0.06, 0.36]
Authority	0.26 [0.10, 0.40]	0.27 [0.12, 0.41]	0.25 [0.09, 0.39]	0.26 [0.10, 0.40]
Purity	0.26 [0.10, 0.40]	0.27 [0.11, 0.41]	0.24 [0.08, 0.38]	0.25 [0.09, 0.39]
Individ.	0.18 [0.02, 0.33]	0.18 [0.02, 0.33]	0.16 [0.00, 0.32]	0.17 [0.01, 0.32]
Binding	0.30 [0.14, 0.44]	0.31 [0.15, 0.44]	0.27 [0.12, 0.41]	0.29 [0.13, 0.43]
**Models built with Combination of Lyrics Features**				
MFT	EX14	EX15	EX16	EX17
Care	0.11 [-0.05, 0.26]	0.11 [-0.05, 0.27]	0.11 [-0.05, 0.27]	0.11 [-0.06, 0.26]
Fairness	0.09 [-0.07, 0.24]	0.09 [-0.08, 0.24]	0.07 [-0.09, 0.23]	0.08 [-0.08, 0.24]
Loyalty	0.18 [0.02, 0.33]	0.21 [0.06, 0.36]	0.20 [0.04, 0.35]	0.22 [0.06, 0.37]
Authority	0.25 [0.10, 0.40]	0.27 [0.12, 0.42]	0.26 [0.10, 0.40]	0.26 [0.10, 0.41]
Purity	0.26 [0.10, 0.40]	0.28 [0.13, 0.42]	0.23 [0.08, 0.38]	0.27 [0.11, 0.41]
Individ.	0.10 [-0.07, 0.25]	0.11 [-0.05, 0.27]	0.09 [-0.07, 0.25]	0.11 [-0.05, 0.26]
Binding	0.29 [0.14, 0.43]	0.30 [0.15, 0.44]	0.27 [0.12, 0.42]	0.29 [0.14, 0.43]
**Models built with Combination of Lyrics & Audio Features**				
MFT	EX18	EX19	EX20	EX21
Care	0.17 [0.01, 0.32]	0.17 [0.01, 0.32]	0.16 [0.00, 0.31]	0.17 [0.01, 0.32]
Fairness	0.12 [-0.04, 0.27]	0.11 [-0.05, 0.27]	0.13 [-0.03, 0.28]	0.12 [-0.04, 0.28]
Loyalty	0.21 [0.05, 0.36]	0.21 [0.06, 0.36]	0.23 [0.07, 0.38]	0.23 [0.07, 0.37]
Authority	0.28 [0.13, 0.43]	0.27 [0.11, 0.41]	0.27 [0.11, 0.41]	0.27 [0.12, 0.41]
Purity	0.29 [0.13, 0.43]	0.29 [0.13, 0.42]	0.26 [0.10, 0.40]	0.27 [0.12, 0.42]
Individ.	0.18 [0.02, 0.33]	0.17 [0.01, 0.32]	0.18 [0.02, 0.33]	0.18 [0.02, 0.33]
Binding	0.32 [0.16, 0.46]	0.32 [0.16, 0.45]	0.30 [0.15, 0.44]	0.31 [0.16, 0.45]
