# Supplementary material for: Soundscapes of morality: Linking music preferences and moral values through lyrics and audio
Source: PLoS One. 2023 Nov 29;18(11):e0294402. doi: 10.1371/journal.pone.0294402 (PMC10686442; doi:10.1371/journal.pone.0294402)
Supplement: S4 Table — (PDF) [file pone.0294402.s004.pdf]

S4 Table

MFT	Model 1	Model 2	p
Care	Best Audio Features	All Audio Features	.358
	Best Audio Features & Baseline	All Audio Features & Baseline	.447
	Best Lyrics Features	All Lyrics Features	.775
	Best Lyrics Features & Baseline	All Lyrics Features & Baseline	.714
	Best Lyrics & Audio Features	All Lyrics & Audio Features	.300
	Best Lyrics & Audio Features & Baseline	All Lyrics & Audio Features & Baseline	.281
Fairness	Best Audio Features	All Audio Features	.115
	Best Audio Features & Baseline	All Audio Features & Baseline	.116
	Best Lyrics Features	All Lyrics Features	.791
	Best Lyrics Features & Baseline	All Lyrics Features & Baseline	.804
	Best Lyrics & Audio Features	All Lyrics & Audio Features	.370
	Best Lyrics & Audio Features & Baseline	All Lyrics & Audio Features & Baseline	.263
Loyalty	Best Audio Features	All Audio Features	.004
	Best Audio Features & Baseline	All Audio Features & Baseline	.007
	Best Lyrics Features	All Lyrics Features	.131
	Best Lyrics Features & Baseline	All Lyrics Features & Baseline	.587
	Best Lyrics & Audio Features	All Lyrics & Audio Features	.010
	Best Lyrics & Audio Features & Baseline	All Lyrics & Audio Features & Baseline	.022
Authority	Best Audio Features	All Audio Features	.003
	Best Audio Features & Baseline	All Audio Features & Baseline	.021
	Best Lyrics Features	All Lyrics Features	.000
	Best Lyrics Features & Baseline	All Lyrics Features & Baseline	.001
	Best Lyrics & Audio Features	All Lyrics & Audio Features	.033
	Best Lyrics & Audio Features & Baseline	All Lyrics & Audio Features & Baseline	.113
Purity	Best Audio Features	All Audio Features	.000
	Best Audio Features & Baseline	All Audio Features & Baseline	.001
	Best Lyrics Features	All Lyrics Features	.000
	Best Lyrics Features & Baseline	All Lyrics Features & Baseline	.004
	Best Lyrics & Audio Features	All Lyrics & Audio Features	.001
	Best Lyrics & Audio Features & Baseline	All Lyrics & Audio Features & Baseline	.004
Individ.	Best Audio Features	All Audio Features	.167
	Best Audio Features & Baseline	All Audio Features & Baseline	.221
	Best Lyrics Features	All Lyrics Features	.762
	Best Lyrics Features & Baseline	All Lyrics Features & Baseline	.712
	Best Lyrics & Audio Features	All Lyrics & Audio Features	.246
	Best Lyrics & Audio Features & Baseline	All Lyrics & Audio Features & Baseline	.202
Binding	Best Audio Features	All Audio Features	.000
	Best Audio Features & Baseline	All Audio Features & Baseline	.001
	Best Lyrics Features	All Lyrics Features	.000
	Best Lyrics Features & Baseline	All Lyrics Features & Baseline	.022
	Best Lyrics & Audio Features	All Lyrics & Audio Features	.003
	Best Lyrics & Audio Features & Baseline	All Lyrics & Audio Features & Baseline	.012
