# Supplementary figures and images for: Soundscapes of morality: Linking music preferences and moral values through lyrics and audio
Source: PLoS One. 2023 Nov 29;18(11):e0294402. doi: 10.1371/journal.pone.0294402 (PMC10686442; doi:10.1371/journal.pone.0294402)

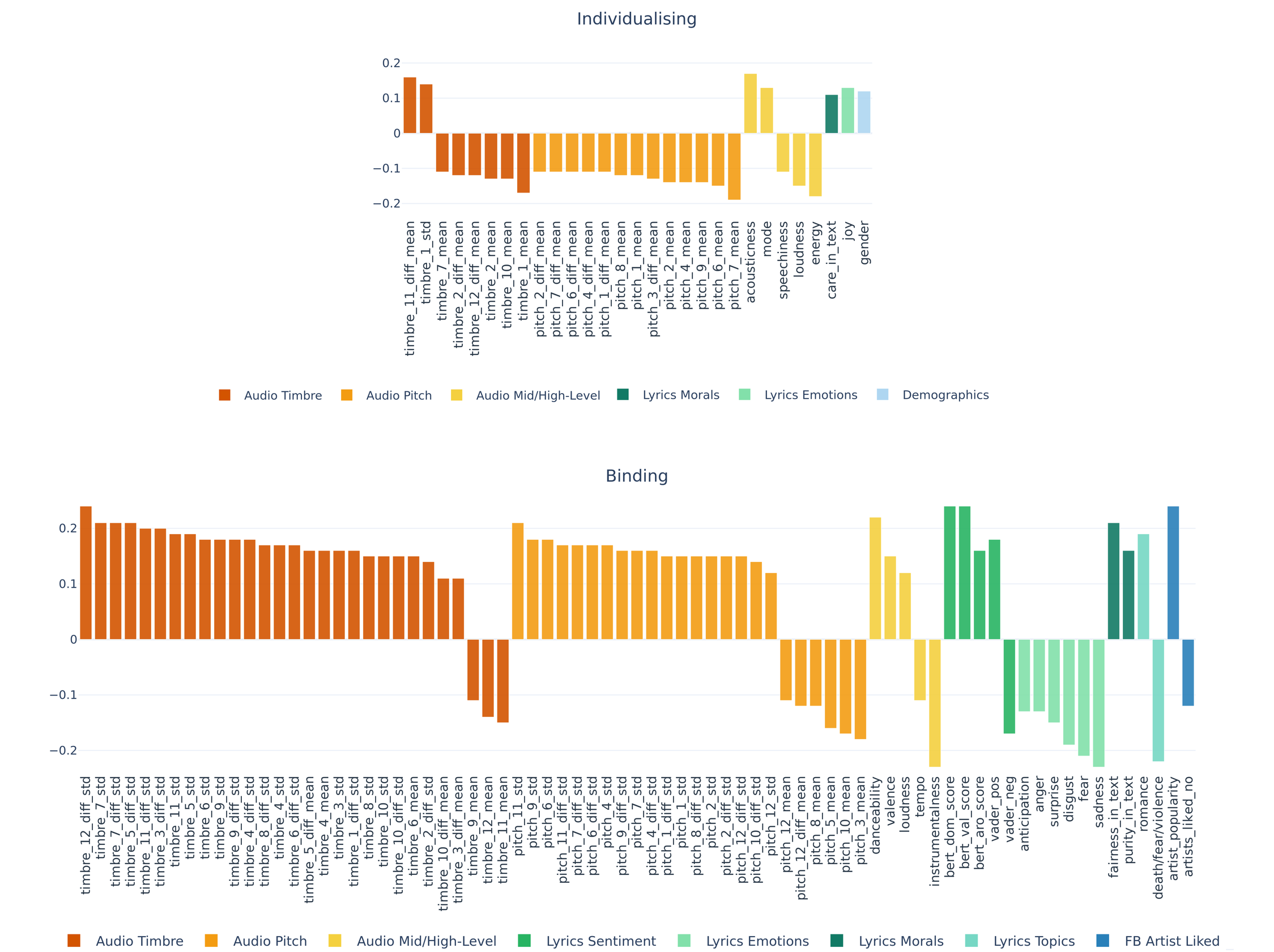

Supplement: S1 Fig — (TIFF) [file pone.0294402.s005.tiff]

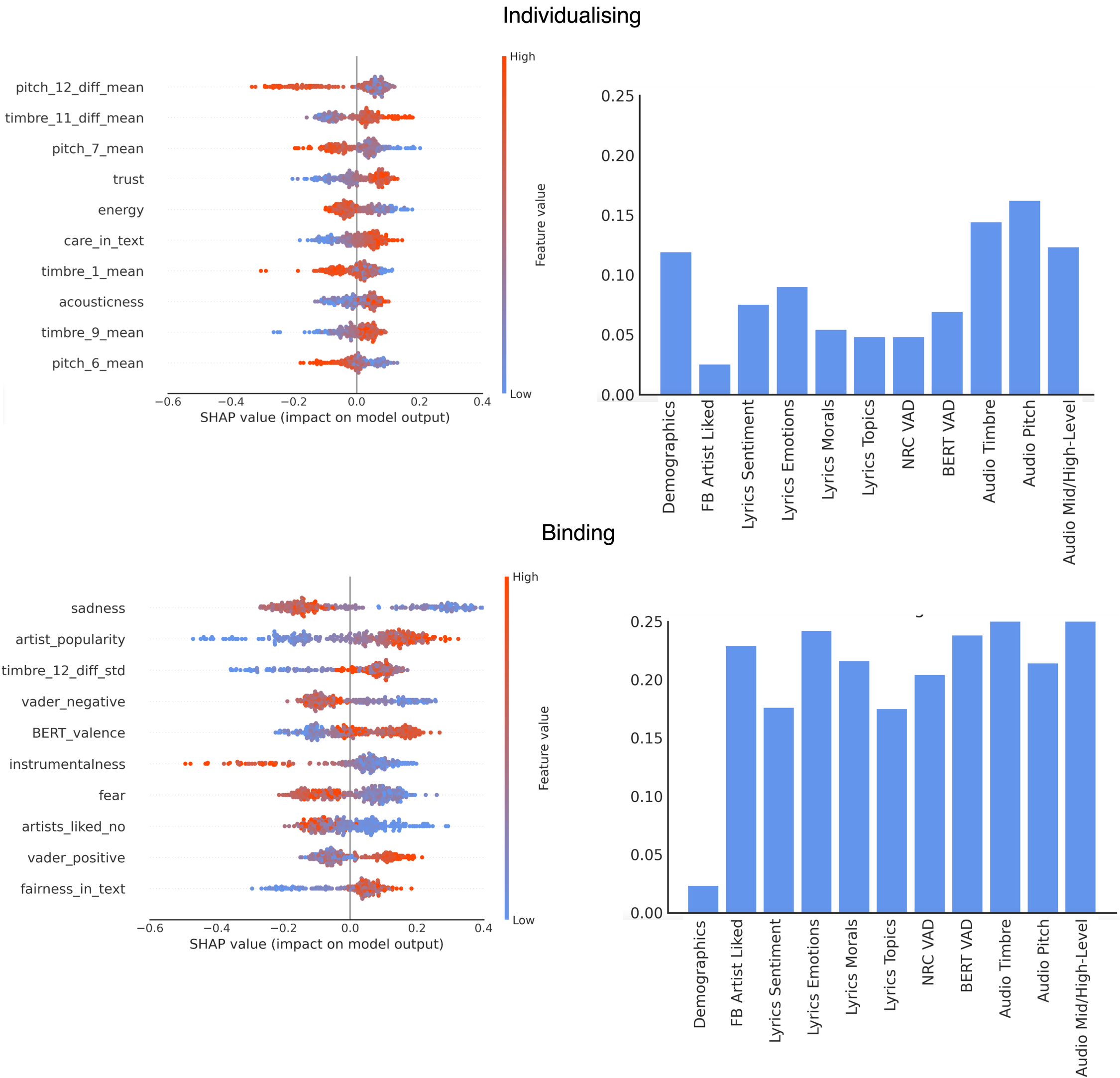

Supplement: S2 Fig — Left: Top 10 SHAP values from the model with best lyrics and best audio features. Right: Pearson correlations between predicted values from regression and actual values for different groups of features. (TIFF) [file pone.0294402.s006.tiff]

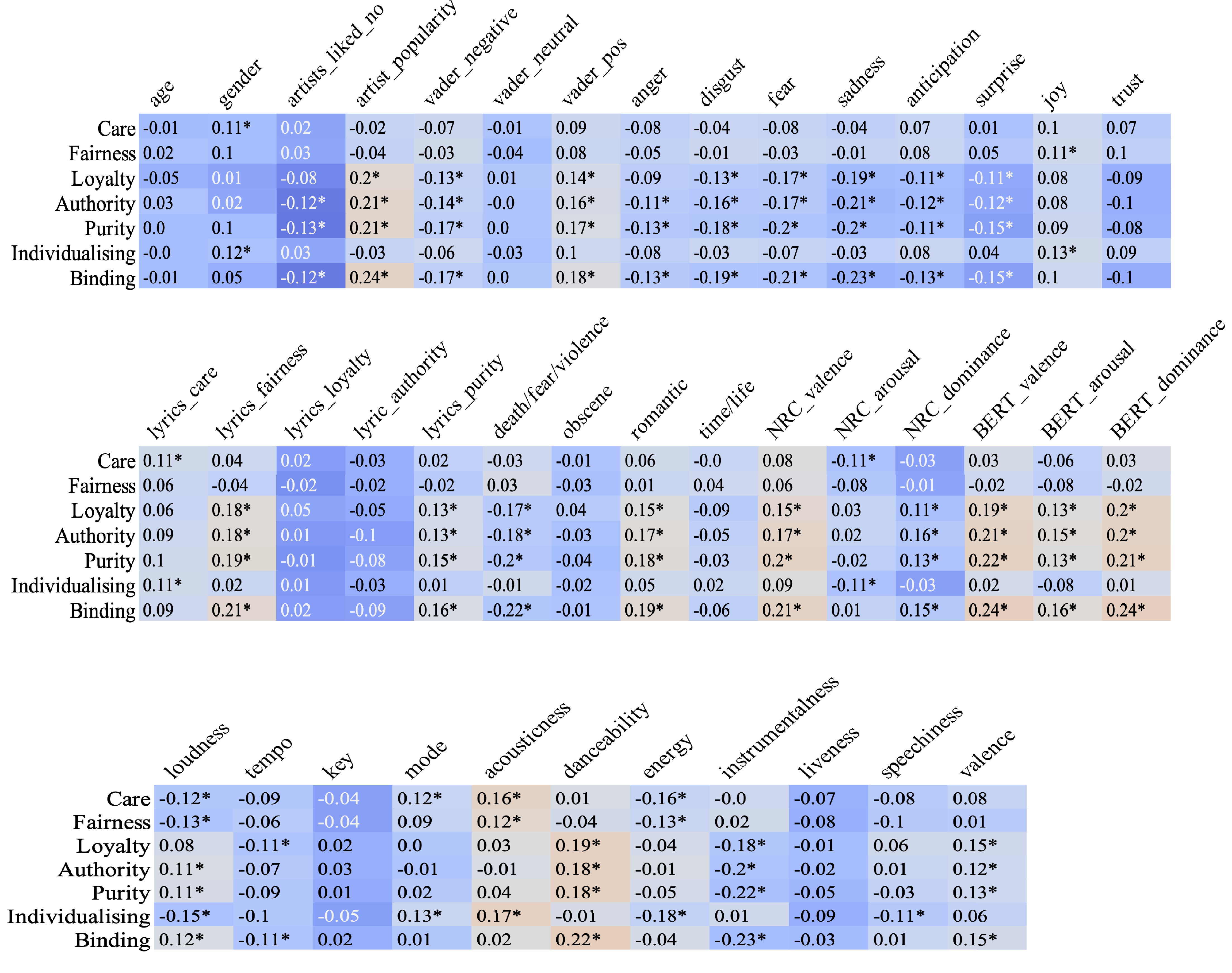

Supplement: S3 Fig — Significance threshold (*) corrected to 5.3 × 10^−5^. (TIFF) [file pone.0294402.s007.tiff]

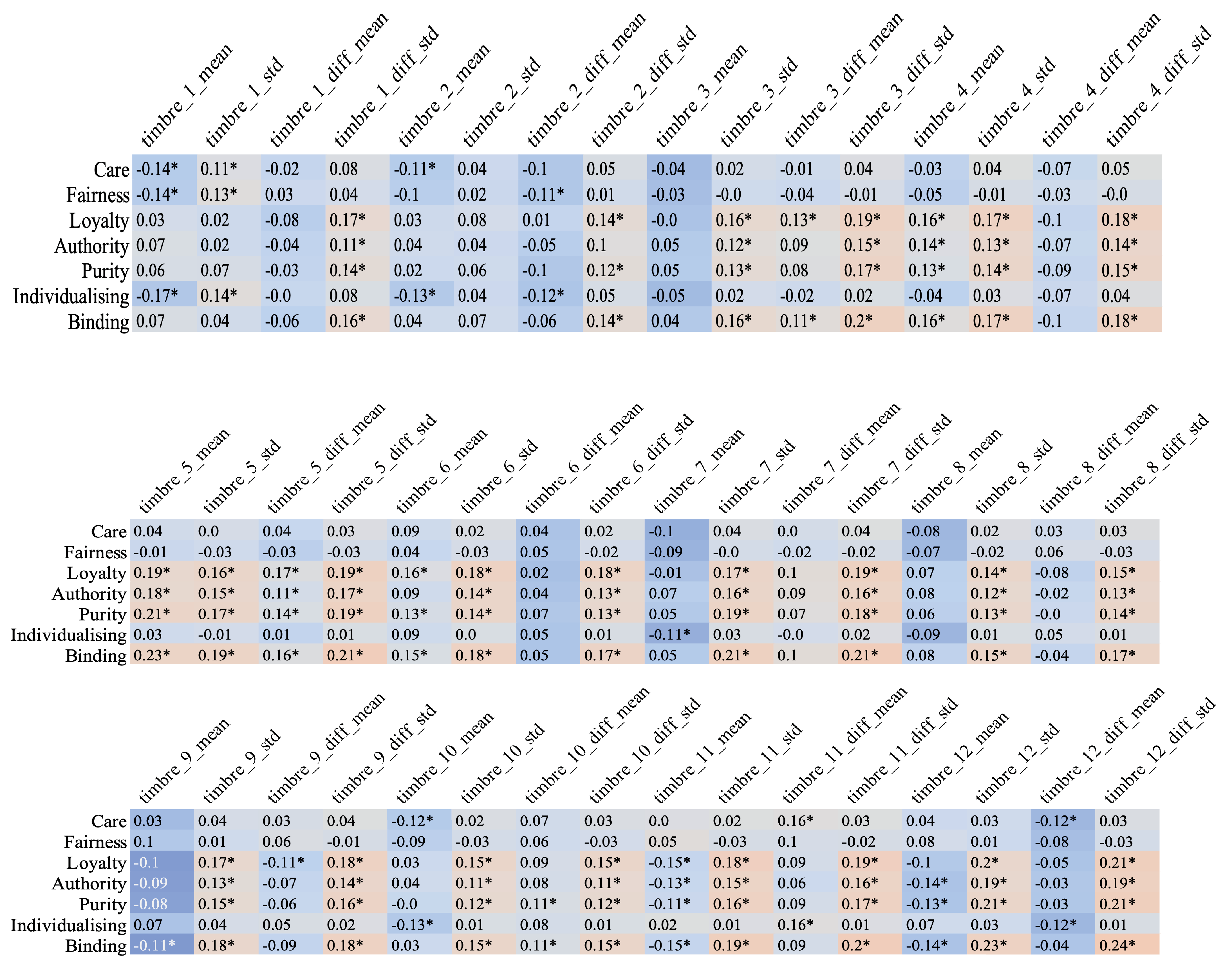

Supplement: S4 Fig — Significance threshold (*) corrected to 5.3 × 10^−5^. (TIFF) [file pone.0294402.s008.tiff]

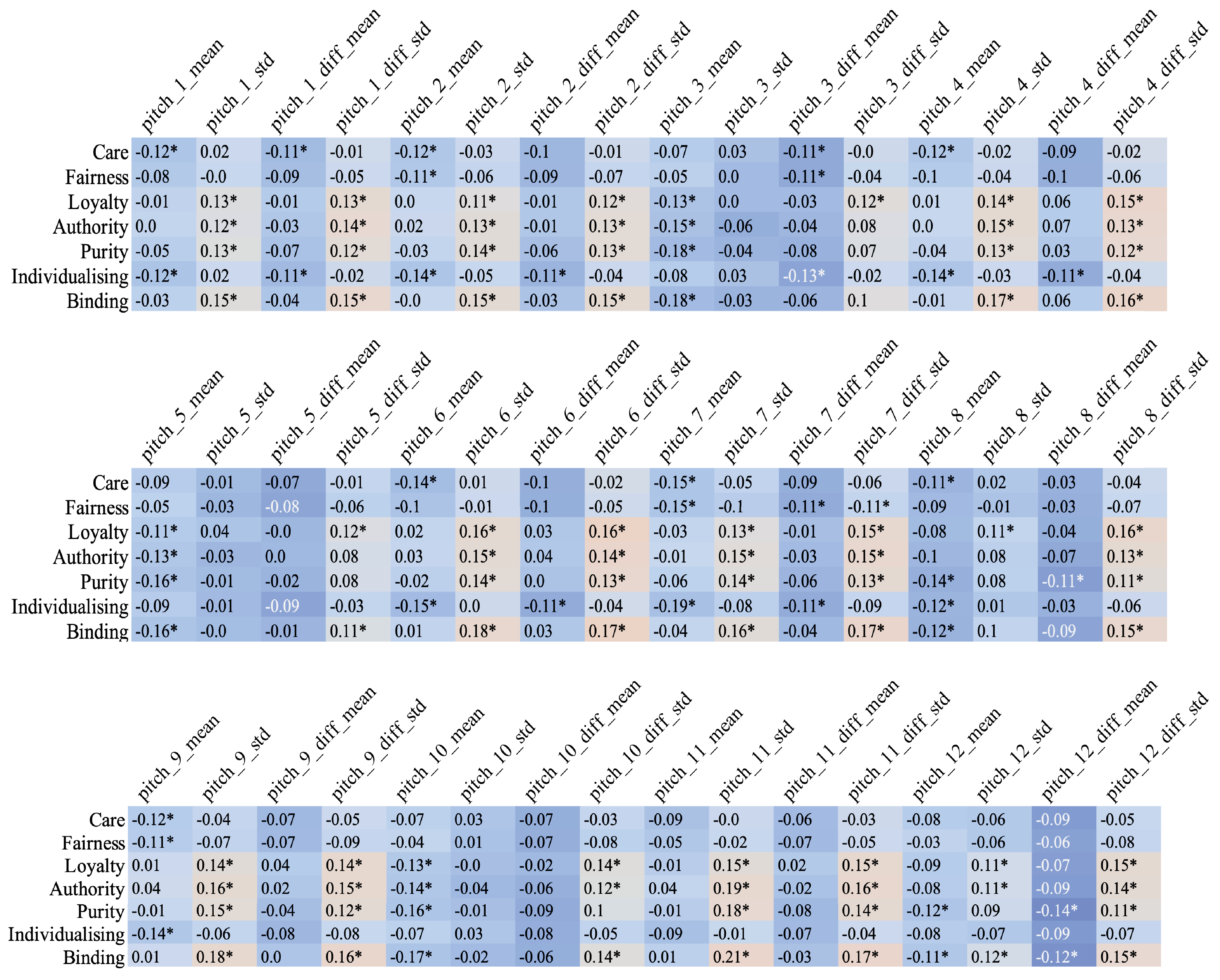

Supplement: S5 Fig — Significance threshold (*) corrected to 5.3 × 10^−5^. (TIFF) [file pone.0294402.s009.tiff]
